# Supplementary material for: Tardigrade secretory proteins protect biological structures from desiccation
Source: Commun Biol. 2024 May 25;7:633. doi: 10.1038/s42003-024-06336-w (PMC11127935; doi:10.1038/s42003-024-06336-w)
Supplement: Supplementary file 2 — Supplementary Information [file 42003_2024_6336_MOESM2_ESM.pdf]

Tardigrade secretory proteins protect biological structures from desiccation

Supplementary Information

Table S1. SAHS proteins used in this study for expression in Figure S2.

Name	Uniprot ID	Host organism	Amino acid lengths (including secretion tag)
RvSAHS1	J7MFT5	*R. varieornatus*	169
RvSAHS2	J7MAN2	*R. varieornatus*	174
RvSAHS3	A0A1D1UKM2	*R. varieornatus*	146
RvSAHS4	A0A1D1UN89	*R. varieornatus*	171
HeSAHS4	P0CU42	*H. exemplaris*	174
RvSAHS6	A0A1D1UJP0	*R. varieornatus*	172
RvSAHS7	A0A1D1UQJ5	*R. varieornatus*	159
RvSAHS8	A0A1D1UJQ1	*R. varieornatus*	173
RvSAHS9	A0A1D1UKG0	*R. varieornatus*	113
RvSAHS10	A0A1D1UJR2	*R. varieornatus*	123
RvSAHS11	A0A1D1URS8	*R. varieornatus*	171

RvSAHS12	A0A1D1UNQ6	*R. varieornatus*	110

Table S2. Protein localization predicted by TargetP software, based on sequences from Uniprot files of Table S1.

Protein name	Other	Secretory	Mitochondrial
RvSAHS1	.0001	.9998	.0001
RvSAHS2	.0001	.9999	0
RvSAHS3	.0004	.9995	.0001
RvSAHS4	.0045	.9946	.0009
HeSAHS4	.0001	.9997	.0002
RvSAHS6	.0009	.9989	.0003
RvSAHS7	.9989	.0007	.0004
RvSAHS8	0	1	0
RvSAHS9	.9995	.0003	.0001
RvSAHS10	.9931	.0004	.0065
RvSAHS11	.0007	.9977	.0016
RvSAHS12	.9995	.0002	.0003

Table S3. Amino acid sequences of the SAHS proteins (after SUMO cleavage).

Protein name	Amino acid sequence	Mw(kDa)
RvSAHS1	APAEGHDDAKAEWTGKSWMGKWESTDRIENFDAFISALGLPLEQ YGGNHKTFHKIWKEGDHYHHQISVPDKNYKNDVNFKLNEEGTTQ HNNTEIKYKYTEDGGNLKAEVHVPSRNKVIHDEYKVNGDELEKT YKVGDVTAKRWYKKSSSS	17.3
RvSAHS4	RPHDESKAQWTGKPWLGKWESIDGTPENWEAFVKAANIPPKDQA LYNGKQKTLLKYWKEAGEDHYHVQTSFPGTEHKMETSFKMGQEG TLSHDGVDLKYVCTEDGEQLITKINIPSKNQETIVTYTATGDDL EQTFTSNGVTGKRWYKKIHA	17.3
HeSAHS4	TGDAPKEWSGKPWLGKFVAEVTDKSENWEAFVDALGLPEQFGRA PVKTIQKIYKQGDHYHHIFALPDKNFEKDIEFTLGQEVEIKQGE HIAKTKYSEDGEKLVADVSIPTKGKTIRSEYEVQGDQLIKTYKT GDIVAKKWFKKVANPTEAPAQAA	17.4
RvSAHS6	RPHDESKAQWTGKPWLGKWESTDKTPENWEAFVKAANIEPKYQS LYSGKQKAIITIYKEGDSHYHAQMTFPGTDHKKEWDFKIGQEGT YSMDGTEVKYVYTENGDQLDSKLNIPSKNTEMTHTYKVTGDELE HIFTSNGATGKKWYKKVNNAV	17.6

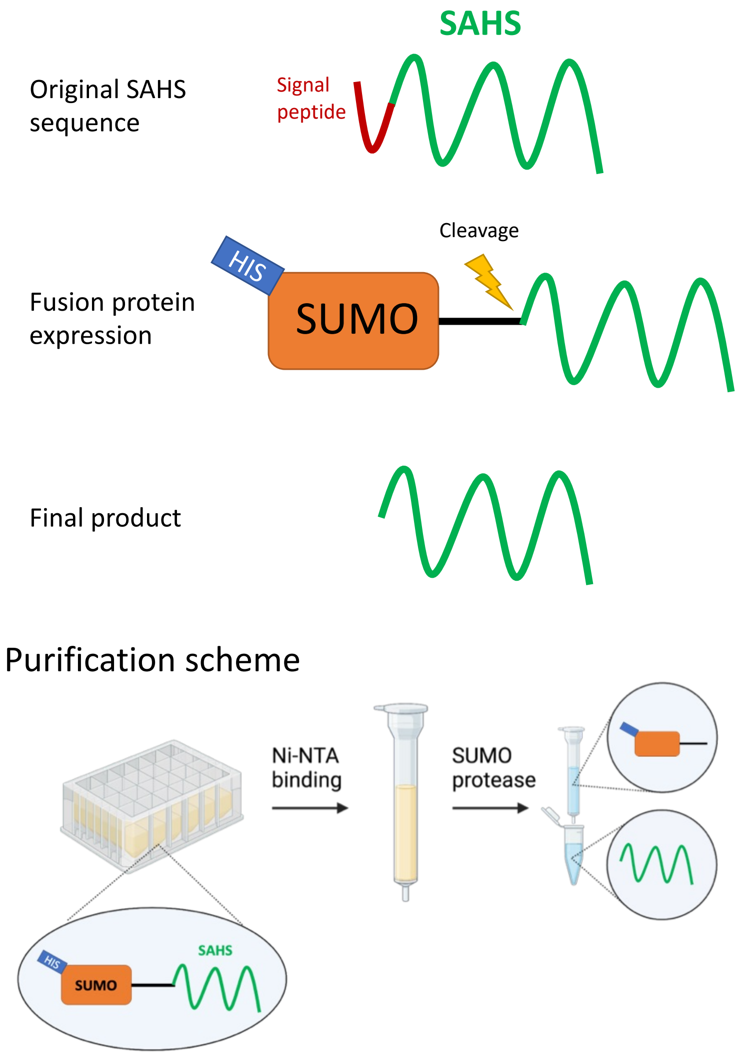

Figure S1. Sequence design purification, and cleavage of the SUMO-SAHS fusion proteins to yield mature SAHS proteins.

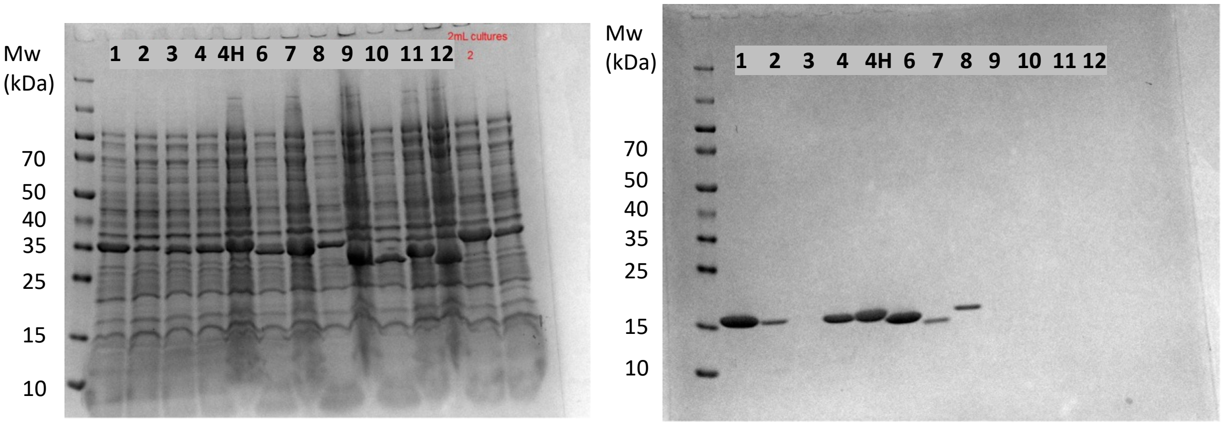

E. coli whole cell

Purified protein (after SUMO cleavage)

Figure S2. Expression and purification of the SAHS proteins from *E. Coli* host. Left: SDS-PAGE of *E. coli* whole cells expressing each SUMO-SAHS construct. Right: final protein products after cell lysis, purification and SUMO cleavage. Each number indicates corresponding RvSAHS protein, and "4H" indicates HeSAHS4. RvSAHS1, 4, 6 and HeSAHS4 were highly expressed and efficiently purified.

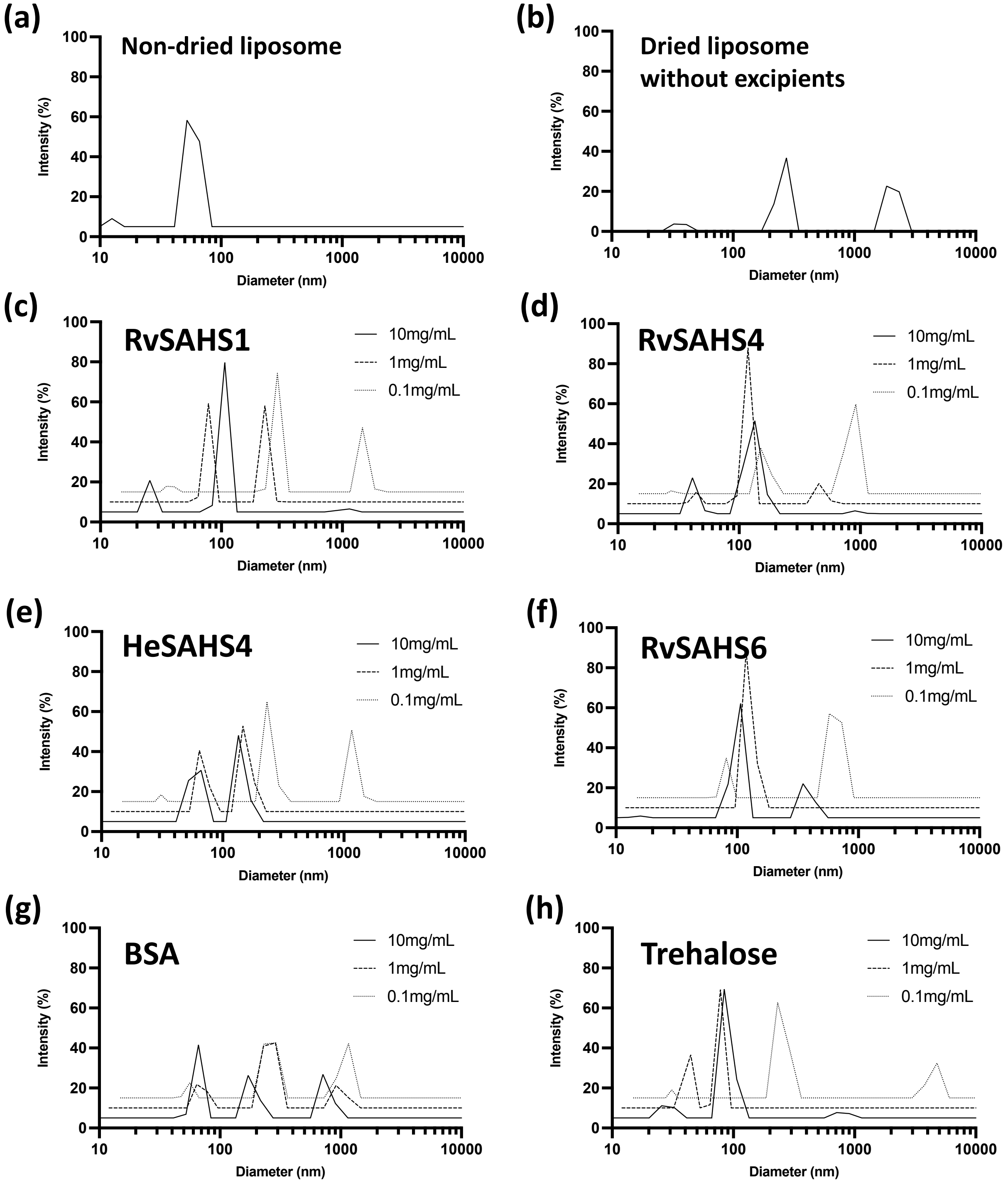

Figure S3. Data from a repeat experiment of that in Figuure 2 showing that SAHS proteins stabilize liposomes from desiccation-induced damage. 1-palmitoyl-2-oleoyl-glycero-3-phosphocholine (POPC)-based liposomes at 1.4 mg/mL were dried with and without the addition

of SAHS proteins or BSA at varying concentrations of 0.1 to 10 mg/mL, and their size distributions were measured by dynamic light scattering. (a) Size distribution of non-dried POPC liposomes (b) Size distribution of POPC liposomes dried and rehydrated without additives. (c-h) Size distributions of the liposomes dried with (c) RvSAHS1, (d) RvSAHS4, (e) HySAHS4, (f) RvSAHS6, (g) BSA and (h) trehalose.

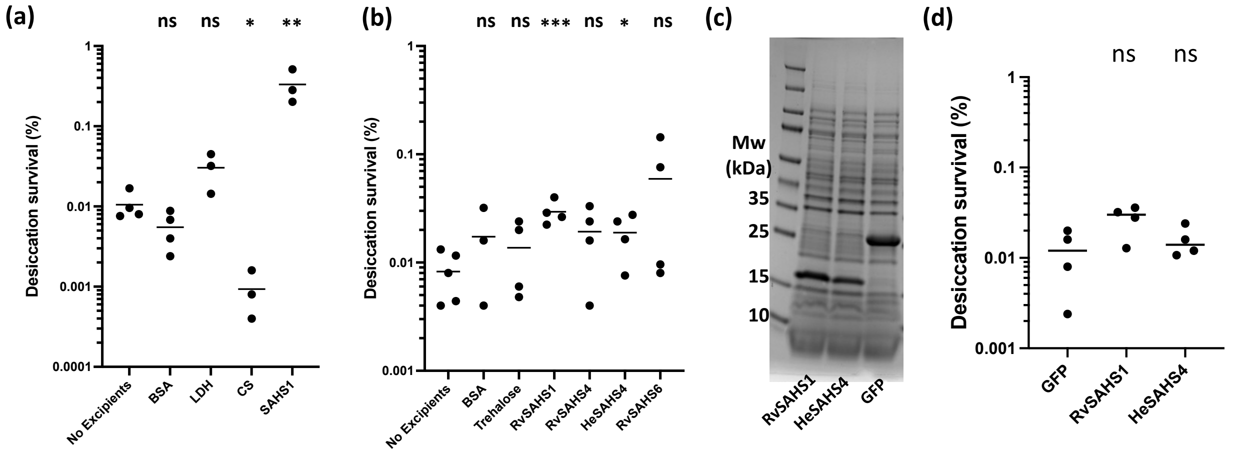

Figure S4. Protection of bacterial cells against desiccation by SAHS proteins. (a) Survival of *E. coli* cells dried for 48 hours with 0.5 mg/mL of BSA, lactate dehydrogenase (LDH) and citrate synthase (CS) as control proteins, and SAHS1. (b) Survival of *E. coli* cells dried with 0.1 mg/mL of extracellularly added SAHS proteins and control excipients. (c) SDS-PAGE of the whole *E. Coli* cells intracellularly overexpressing heterologous RvSAHS1, HySAHS4 and GFP. (d) Survival of dried cells intracellularly expressing each protein. Individual data points represent independent replicates and lines represent the mean survival. The student's t-test was used to determine the statistical significance between the negative control (no excipient) and each group, which is indicated as asterisks. * $p < 0.05$; ** $p < 0.01$; *** $p < 0.001$.

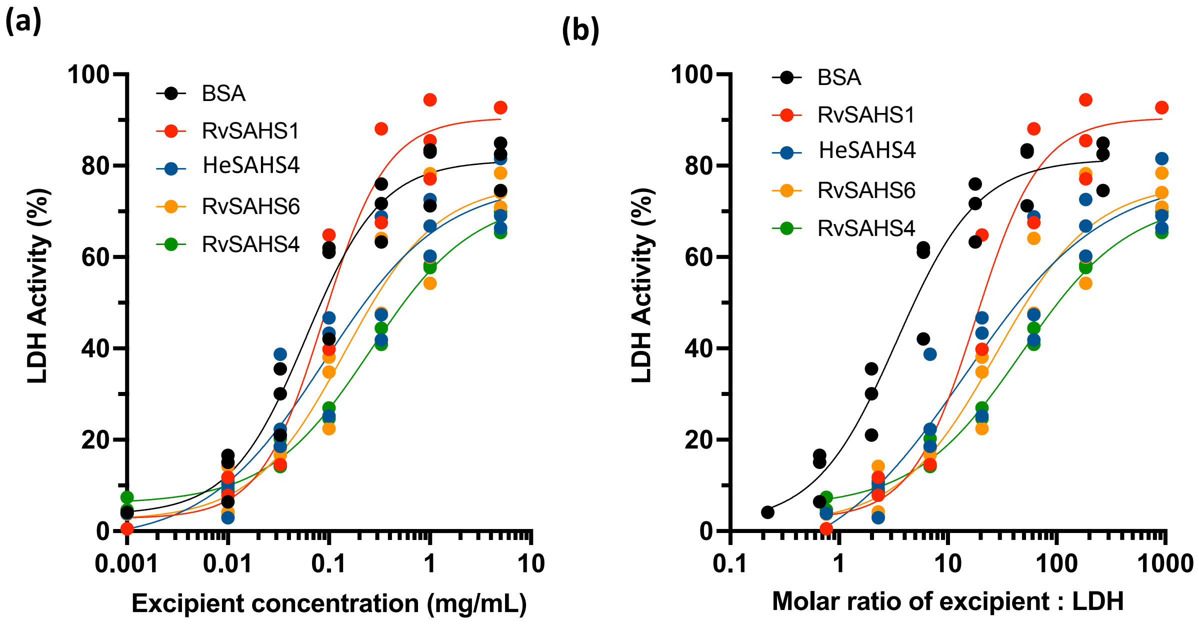

Figure S5. (a) Tardigrade SAHS proteins and BSA protect lactate dehydrogenase (LDH) enzyme activity against drying. LDH (0.01 mg/mL) was desiccated and rehydrated in the presence or absence of SAHS proteins and BSA. Percent activity was determined using non-desiccated control samples stored at 4ºC as the reference to compare activity. (b) The data from the same experiment as (a), using molar ratio between protein excipient and LDH as the x-axis.

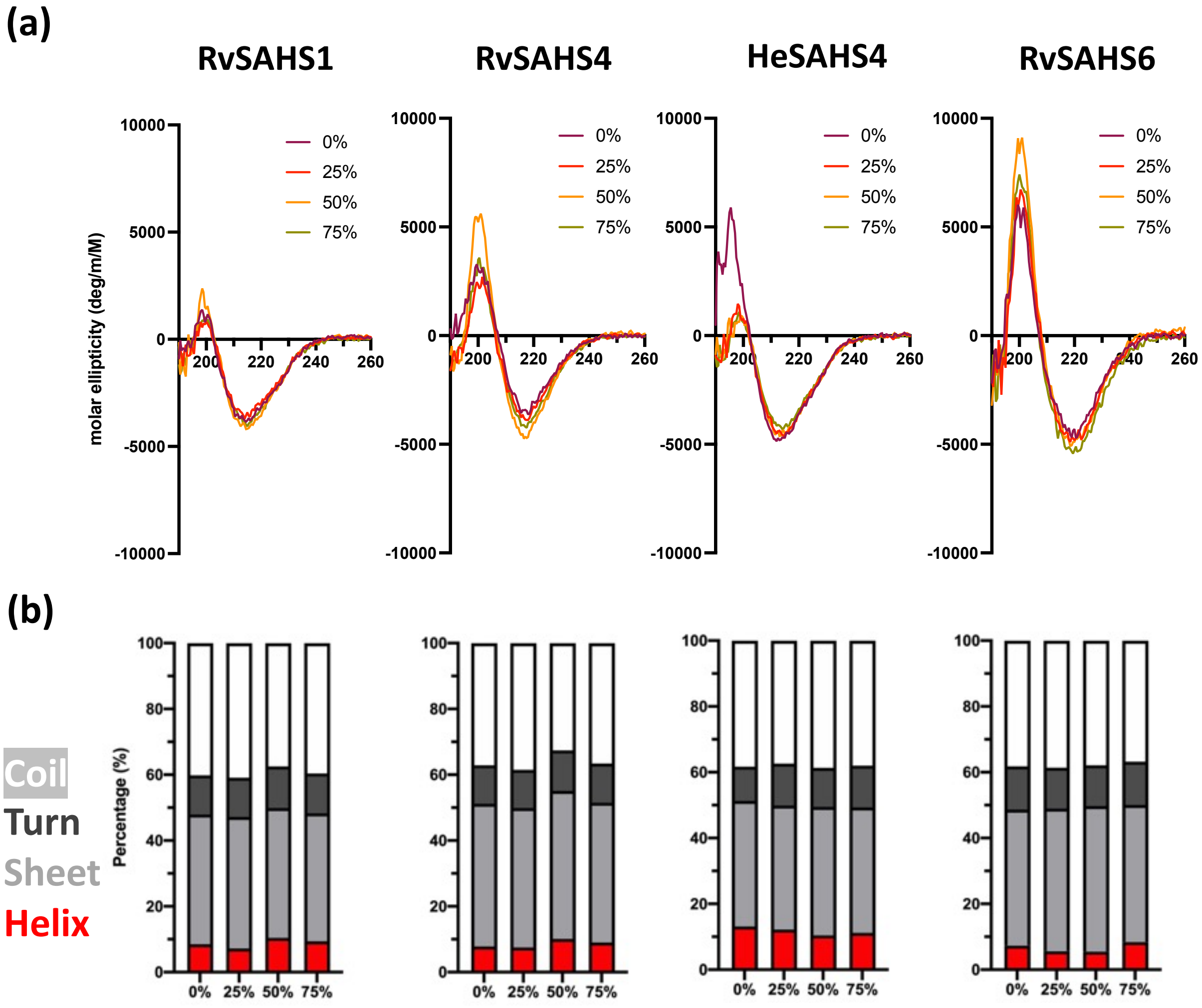

Figure S6. Effect of glycerol on SAHS protein structure. SAHS protein secondary structures upon glycerol addition were determined using circular dichroism. (a) CD spectra of SAHS proteins upon addition of increasing amounts of glycerol from 0 – 75%. (b) Secondary structure compositions of SAHS proteins under different glycerol level, calculated from the CD spectra.

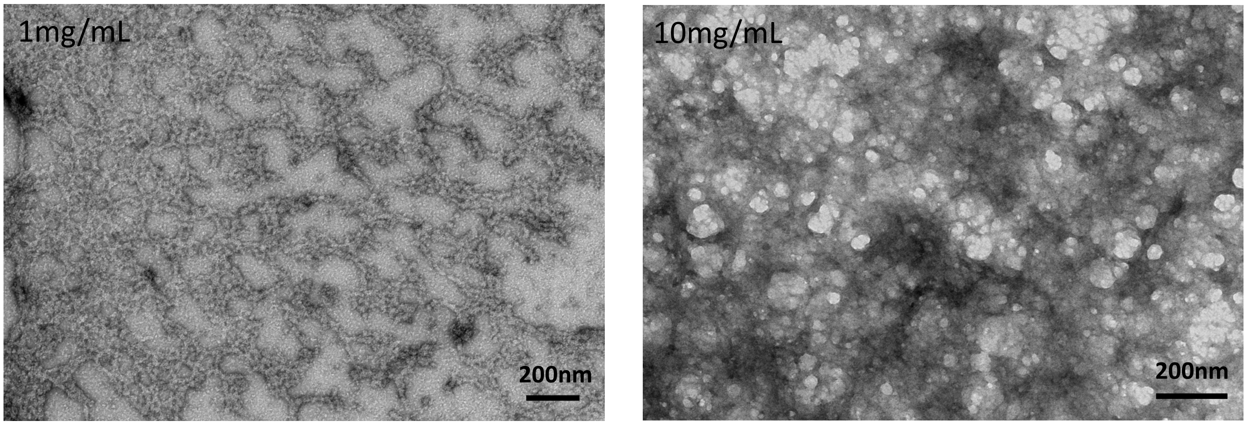

Figure S7. Transmission electron microscopy images of the fibrous network structure formed by RvSAHS1 proteins dried at 1 mg/mL (left) and 10 mg/mL (right) concentrations. Scale bar = 200 nm. Previously, TDPs belonging to the CAHS family have been shown to form filamentous structures upon dehydration both *in vitro* and *in vivo*, and these proteins may adopt increasingly helical structures with loss of water [1, 2]. It is possible that although structurally distinct, SAHS and CAHS proteins share an ability to form higher order structures under dry conditions.

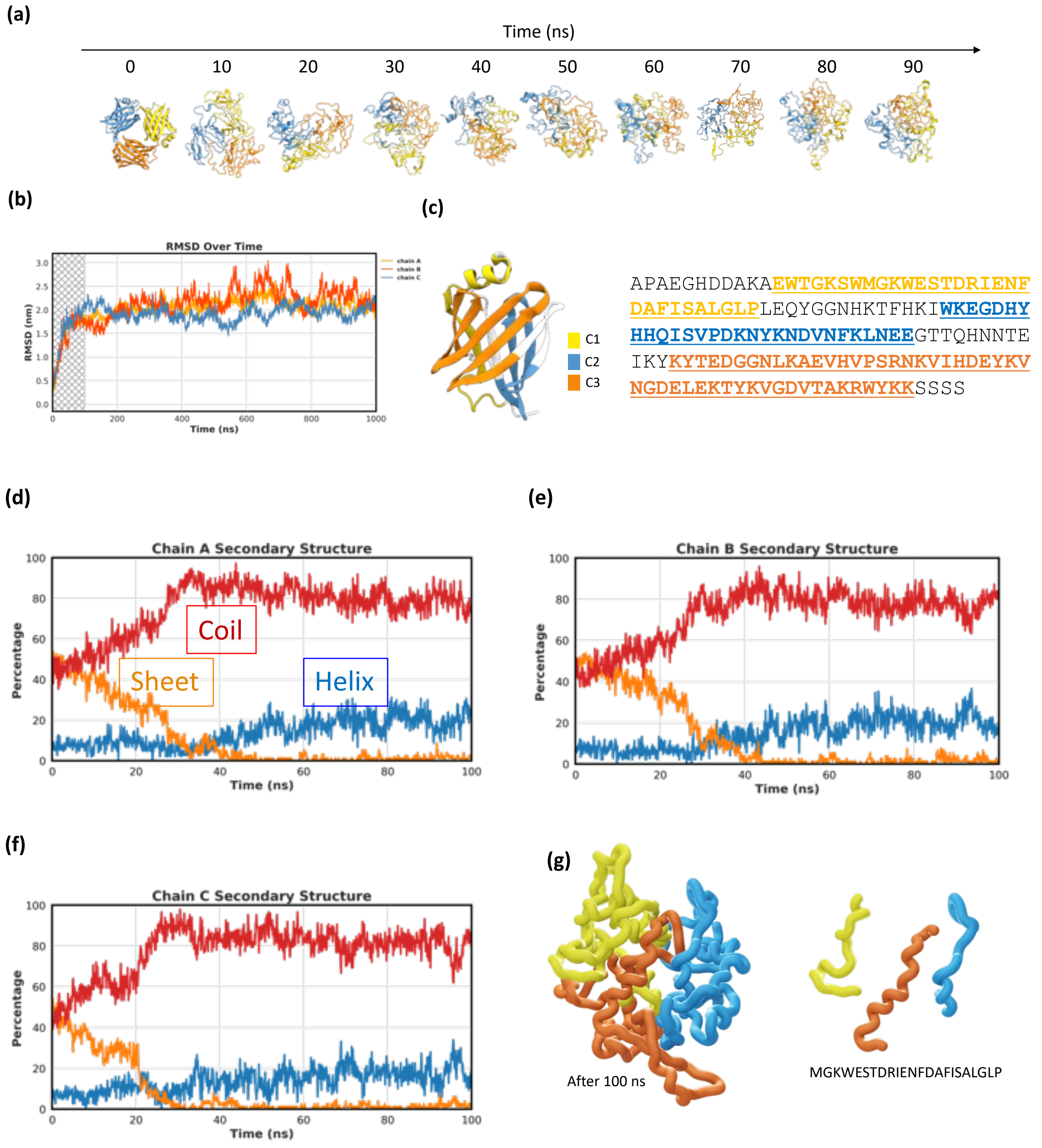

Figure S8. MD simulation of an RvSAHS1 protein trimer. AlphaFold was used to predicted the structure of three SAHS1 proteins, giving the T=0 structure in (a). This structure was used as a starting point for a molecular dynamics simulation for 1 microsecond. (a) Changes in the structure of the RvSAHS1 trimer during the first 100 ns of simulation. (b) Root mean squared

deviation (RMSD) relative to the starting structure over time during the entire 1 microsecond of MD simulation. Different colors indicate each monomer chain in the trimer. (c) Mapping of conserved segments C1-C3 of the RvSAHS1 sequence as defined by Yamaguchi et al. [3] prior to the solving of the SAHS1 structure. Amino acid sequence of RvSAHS1 is indicated, with C1 motif represented in yellow, C2 in blue, and C3 in orange, respectively. (d-f) The distribution of sheet, helix and coil conformations of amino acid backbones for each of the three SAHS1 proteins in the simulation. The sheet/helix/coil classification is based on the psi/phi dihedral angles of each amino acid, and not on hydrogen-bonding patterns that may or may not be present. (g) State of the simulation at 100 nsec (left), and a segment from each of the three SAHS1 proteins represented to illustrate the formation of short helical segments. During the course of the high-temperature simulation, helical segments throughout the proteins are unstable and present transiently.

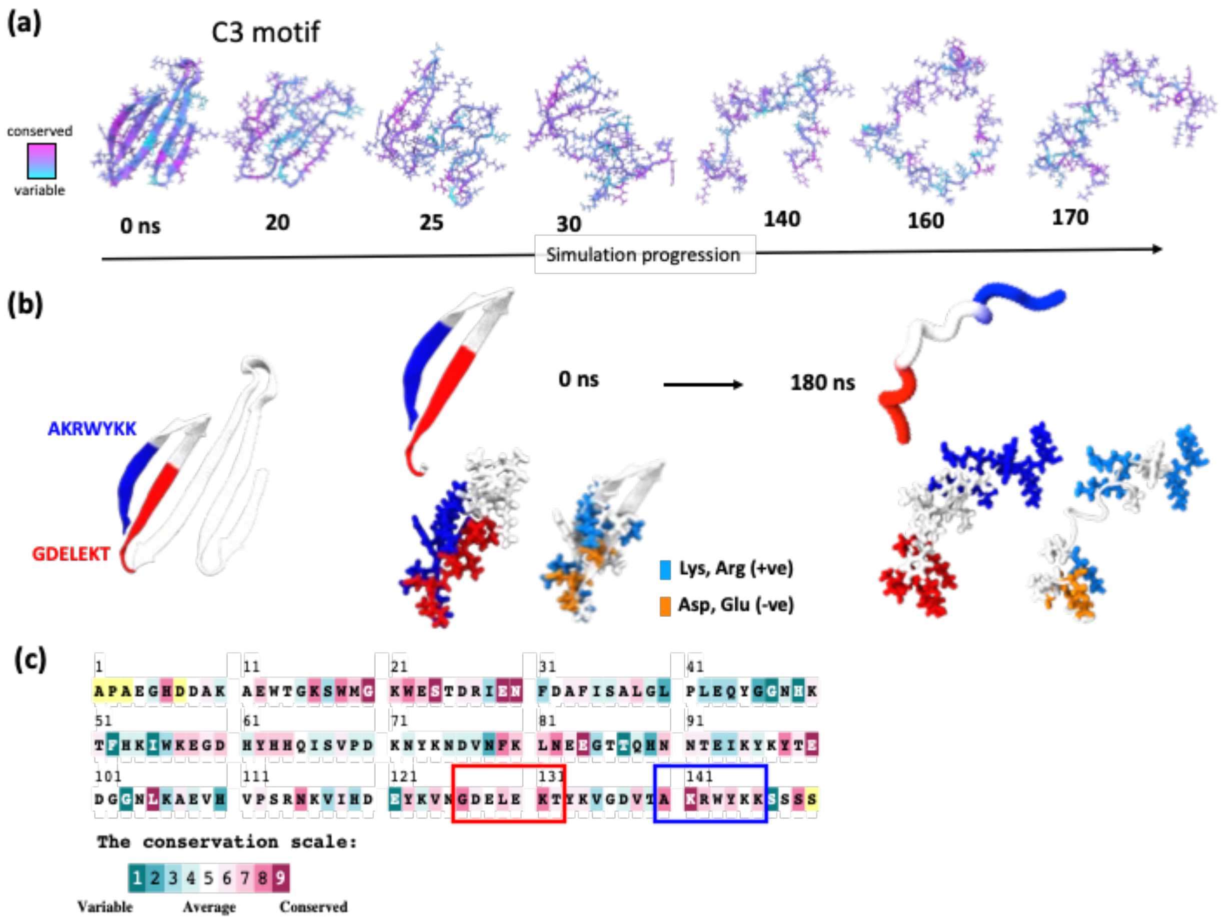

Figure S9. MD simulation showing structural changes in the conserved C3 motif of RvSAHS1. (a) Representative structural changes in C3 motif during the first 170 ns of simulation. Color scheme indicates the degree of evolutionary conservation, which shows that this motif is highly conserved among the SAHS family. (b) Close-up representation of the C3 motif sheet-to-helix structural change. Highlighted in blue and red are two highly conserved regions found within the beta sheet region that directly interact through ionic bonds. Structures of these regions at 0 and 180 ns are indicated, along with an additional depiction of the same regions in which positive residues (Lys, Arg) are highlighted in cyan and negative residues (Asp, Glu) are highlighted in orange. (c) Evolutionary conservation analysis of the RvSAHS1 sequence. Red and blue boxes indicate the same sequences depicted in (b).

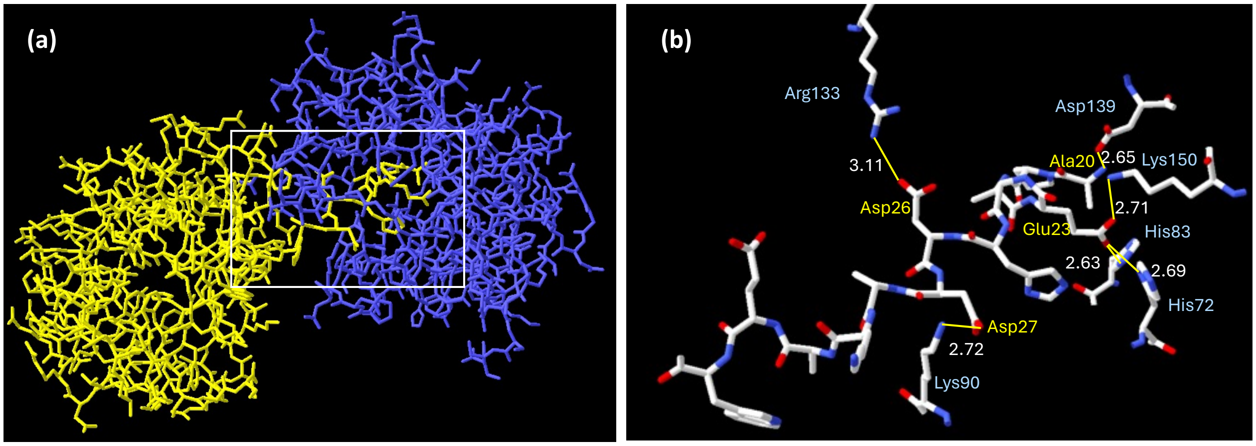

Figure S10. AlphaFold-predicted insertion of the SAHS1 N-terminal tail into the SAHS1 cavity. The AlphaFold-predicted structure of three SAHS1 proteins indicated that the N-terminal ~9 amino acids of mature SAHS1, which are predicted to be unstructured in a monomer [4], might be able to occupy the cavity of a second SAHS1 protein. The T=0 nanosecond structure in Figure S8(a) shows the trimeric structure predicted by AlphaFold, which contains three different insertions of an N-terminus into the cavity of an adjacent SAHS1 protein. These differ in detail, and we regard the complex in this figure as the most structurally plausible. (a) A SAHS1 protein (yellow) with an N-terminal tail located in the cavity of a second SAHS1 protein (blue); this is a close-up view of two of the three proteins depicted in Figure S8(a), T=0. (B) An expanded view of the region in the box of panel (a) in which the first 14 amino acids of the yellow subunit are shown (Ala20-Trp32, with interacting amino acids lettered in yellow) and select interacting amino acids from the blue SAHS1 protein shown and lettered in light blue. Also shown are the distances, in Angstroms, between oxygens in one side chain and protonated nitrogens in another, in cases of possible hydrogen bonding. Amino acid numbering is relative to the first amino acid in the translated sequence (as in Figure 1), such that after removal of the 19-amino acid signal sequence, Ala20 is the first amino acid in the mature SAHS1.

We note that when Fukuda et al. solved the structure of SAHS1 [4], they removed the N-terminal 10 amino acids of the protein because this segment might interfere with crystallization, and the cavity was filled with solvent molecules. The crystallized form of SAHS4 included an extra serine at its N-terminus, which may have disrupted insertion of the N-terminus into the SAHS4 cavity, given the tight packing and specific contacts made by the N-terminal amino group in the SAHS1 model of panel b [5]. The human liver and heart muscle fatty acid binding proteins, which are otherwise similar to the SAHS proteins and possess a large cavity, lack these N-terminal tails (see Figure 1b).

Supplementary Discussion: sequence annotation of SAHS9, 10 and 12.

The "short" SAHS proteins, SAHS9, 10 and 12 from *R. varieornatus* are truncated at their N-termini relative to other SAHS proteins. The Uniprot versions of SAHS9, 10 and 12 lack signal sequences, could not be expressed in a soluble form, lack residues that appear to be important in forming the hydrophobic core in SAHS1 and SAHS4, and for SAHS9 and SAHS12 began with a methionine corresponding to a methionine also found in SAHS3, 7 and 8. We therefore explored whether these might be incorrectly annotated and examined the genome sequence of *R. varieornatus* for upstream coding sequences that might have been overlooked. The results of this analysis are that the SAHS9, 10 and 12 genes encode proteins that may extend in the N-terminal direction further than the annotation would indicate, and that the new predicted protein sequences align well with N-termini of other SAHS proteins. However, we have not found clear-cut start codons for *SAHS9* and *10*, and SAHS12 may be much larger than the other SAHS proteins. The annotation below is intended to be a work in progress.

The *SAHS9*, *10* and *12* genomic regions were obtained from Genbank accession # *BDGG01000001* (contig 1), a large contig from the *R. varieornatus* genome [6]. Relevant genome sequence fragments were obtained as reverse complements, then copy-pasted into Microsoft Word, and visually scanned for the presence of splice acceptors upstream of putative start codons for ORFs that translate into the Uniprot sequences. Because all of the SAHS genes in *BDGG01000001* are on the anti-sense strand, we generated the reverse complement of this ~ 9Mb sequence for presentation purposes. The numbering used below is according to this reverse complement. Upstream regions of *SAHS9*, *10* and *12* were also copy-pasted into the Expasy Translate Tool (https://web.expasy.org/translate/), translated, and checked for long ORFs. A visual scan of the translated sequences revealed a pattern of tryptophans and other amino acids

that align well with the N-terminus of other mature *SAHS* genes (Figure 1B), but which are not found in the annotated sequences of these genes.

We also examined the intron/exon boundaries of these SAHS proteins and compared them to regions encoding other SAHS proteins to further validate the proposed mature amino acid sequences.

SAHS9 **analysis**

The SAHS9 protein sequence is most similar to SAHS10, 2 and 8. *SAHS9* is also adjacent to *SAHS2* and *SAHS8* in the genome and is in a ~30 kilobase region that codes for *SAHS11* plus *SAHS10* (also adjacent) and *SAHS7* plus *SAHS1* (also adjacent).

When the *SAHS9* genomic sequence is aligned with, for example that of *SAHS2*, there is a putative splice acceptor in *SAHS9* DNA upstream of the annotated start codon at the same position as a splice acceptor annotated in SAHS2. Moving upstream, there is a putative splice donor in *SAHS9* such that an intron of 79 bp is predicted, similar in size to the 82 bp intron in *SAHS2*. Moving further upstream, there is a predicted exon encoding a protein segment that aligns well with other full-length SAHS proteins, including Trp residues that help define the hydrophobic core. However, the predicted splice acceptor upstream of this exon has the sequence . . . TCCTTCTTACCG, lacking the canonical AG, and yet further upstream it is difficult to identify a splice donor and start codon that would align with other *SAHS* genomic sequences. It is possible that *SAHS9* is a pseudogene, that this region of the sequence contains one or more errors, or that some other mechanism operates to express this gene.

Below is the genomic region encoding *SAHS9*, *2* and *8*.

```
2575501 atttttcgcc tgtacagaca taagctgtta gagctgatgg agacacacct agatctcggg
2575561 ttgacaccca atgacacggt aatgatcctt agaccattgt ccgaaaaatt gctcatcacc
2575621 gttccctgta tggaaaaaaa ctggcttggc gggtaccttc gtgaacaata gtatgtcagt
```

```
2575681 tgagttttcg gtgagtcata aggggcagat agccgcgtgg cggaagcagc tgtttcggcc
2575741 tgtactgcaa accatgcata ggaaattggt ttaaaaaacg acttttccag ttttcttccc TATA box
2575801 tacatggata tttccgccca aatttagcca tccattcgtt tgtagaatgt ttccagcatt
2575861 cgataccttta aggcatgctc ttgataacta cataagcggt ttcaagagtt cgactaacgc
2575921 ccctccagaa aacgtcgttt cctgaacagt ttagaaaata cctcggaacg gccaattaga splice donor
2575981 aaaaaacagg cgtcagttat gctgtacatt gctgcaacgg tgtacaggtc gaccgagcca
2576041 acggaatccg aataacatcc gaataatatc cttcttaccg gcgctgccat cgccattctc end of signal
2576101 atcgaagacc ctgctgacga aaaaggagca gaatggaccg gaaaaccgtg gctgggcgaa exon 2 Trp codons
2576161 tgggtctctg tacccgagca ggacaaaaac ctggcacagt tcaagaggaa gcttcgtaag
2576221 ttttacgtct cctgtgtgac tttgatcttt ctgtacgatt gatgacgttc ttcccgaata
2576281 tatatcgttc acagagctgc ctatgagcca tccggaagtc aatctcaact ctactgtctt SAHS9 “start”
2576341 ggtcaaccac ctcaagaagg gagatgaata ccatcacaag attatcatca aagaatatta exon 3
2576401 caccaatcac gtaagtttat aaccgttagc gcgctcttaa aaaaagcaaa atggtcacgc
2576461 gaaaacgttt ttcaggtcgt ttacaagctg ggcgagcagt cacccggctc gtacgacggt
2576521 ttgtcctata gtgtaaagta tggagagaaa gatggcgcgt tggttggaac ggcccattac exon 4
2576581 acaggcacca aagaccagcg tctcaacata accatgcaca acgtctacaa gctcgaaggg
2576641 gatcgtcttc tcaagagctc caccatcgac ggagtaacac tgaattgcca tcacaagagg
2576701 cgcatctgaa gctgtgcacg tcgtttcagt tcttcgactt ttatcgatat tcttatagtc
2576761 ttgatcaacg cttgagtaaa ggtgtttcaa gatgcataaa gactttcttc gtccttggcg
2576821 aagcttatca gtcatcttct cgaatcaaga tatccattcc atcggcagaa ggagagattc
2576881 tccgctttcc acacttgcgt gggtagactc gtgtagagcc gcgcgtatcg cggtgaacat
2576941 cgagaaaggt tcgaagcttg gcttagagtt ctcagacggt cacgggacaa gatcagatac
2577001 ggcgcaaggg tgttttcccc agcatgcaga ccgttatgac acatagttga ctttgatgcg
2577061 actggtggta ctgggaagaa tgtaacgctc caatattcgc ctaaggtgta atcctaaggt
2577121 atacgtacag tagacgcaaa cagtacagac tcctccagca acggtacttc ctggccgtat TATA box
2577181 ataaaatcaa ggttcgggtt ccttccaacc tgtacctctt tacttcttgt aactttctca
2577241 cggagaatac tcgcagaacc atgcatcgat ttgtccttgc tctcgtcgtt tttgccggta exon 1
2577301 aacttgaaag cttcttaaga gatttaccgt cttgtgtgct atgaaagagg acgagcatga Intron 1
2577361 gctaaaaggt catcgtaaga aacgtgcgtg gctatatatg tacattttct cctggctttg
2577421 caggtgctgc catcgtctgg gccgctgatg acgctgctca cgaagaaggc gtagaatgga signal sequence
2577481 ctgggaaacc gtggatgggc aaatggggaat ccgacccatc gaaggacgag aacgttgagg exon 2
2577541 aattcaaaaa gaagctccgt aagttacttt gcatttgcat ctcctcgtta gttagttttt Trp codons
2577601 ttagtgttca gtcgacgctg gttgtctggt ggattcgtcg aattccccgg cctgatattt
2577661 tttgcatagc tttatagatt tacccgttca gatccgtaca agcgagaaac caagacacct
2577721 atgtttgttt gcagagcttc cgatgagcca ctcggaaatg aacaaaaact ccaaagtttg
2577781 gatccatcac tacaagaagg gagacgagta ccatcacaaa atcatcatca acgacgccca exon 3
2577841 ttacaaaaac gatgtaagtc cgcaaacttt cccggttaca ttgtttctta cgtctttgct
2577901 gcacagatca aagaaaatta ttttcggctt tgtagatcgt cttcaagctg ggtcaagagt
2577961 ccgccggttc gtataacggc tcatctttca gcgtgaagta cgaggacaaa gacggcgctc exon 4
2578021 tagtcggaag cgtccactac actggcacca aagaacagtc tcttgacaag accatcaaca
2578081 acgtcttcaa gctcgaaggt gaccatctgg ttaagacttc caccatcgag ggagtgacca
2578141 tgaagcgcca ctacaacaaa cgccagtgaa gttgtcgttg cggctaaatt ttttcctttc
2578201 tgcaaattca tgcccgtttt gtcgagtctc tcctgcttcc catcgttcta aagatttttg
2578261 cagtactgag ttatcagggc tttgtttctg ttctcgttct atcctcgtat tttcttttcg
2578321 ttcaccggat acagtaaagc tgcgtttcaa agccaggttt tttatctgcc tgttggtcgg
2578381 acggattgtc ggaccaactc agatatcgat cggcctgatt gtaaacagat actacgtatt
2578441 ttctcgtact cttcgcactg gctaacgtta ggttatacgc tctaaacggt gatcgagaaa
2578501 gttttaaaag caggcacgac atactttaaa ttctcaaacg aacagtactt ttcccatcag
2578561 atgaagcata aagcgggttt tcccagcaaa gacgcttcag cccatggcca attgaccttt
2578621 ggccgaaggc tgctggctac gaaagcgctg agtaatgata agtactcgta ctctattgtt
2578681 caactaagaa acctccgaat gtaacccaaa ggagaacgac ttcgtgccaa cttgatctta
2578741 gactgcagaa taaaccagta taaaatcaaa ccttcgccat ttcacagtac aaagcacgtt TATA box
2578801 ttaaactttt ccagccactt tcttcacgta gaagtcgagc cgcaagcatg aagtgtatcc SAHS8 ex.1
2578861 tagctctcgc tctgtttgcc ggtacgtcga cagacaatta aaattttggt gctttttagt
2578921 atcatagggt aatgcaagac gttgactcga cttgtgtgga tgatcacatg agcatgtgat
2578981 tcatagttga tttcattggt ctgttgcagg tgttgccgtc gtgtgggctg gcgatgatgc
2579041 cgctcatgaa gaaggagtcg actggacctc caagccttgg ttgggcaagt gggaatctat Trp codons
2579101 cccggagaag gatgaaaacc tcgtggagtt tctcaagaag ctcagtacgg tgtatagctt
2579161 tctcttcctt cagtattttc tgttctggac acatatcata aacagggtcc tcgctccagt
2579221 ccccgtccac cttagtctta gaaaatttag agttttcgag ctttacttct gttcacagat
2579281 gttcccatgg accactctaa aatgaacgcc accgtcaagg tccacctcaa ccactacaag exon 3
2579341 aaaggagacg attaccacca caagatcatc gtcaaggagg ctgagtacaa gaacgatgta
2579401 agtttgaccg ctttcgatga gttgacccgt cagctagatg accccttttcg gaagtctatc
2579461 ctatggggtt tccgactgac actagacacg taatatctgt ttcgattttt aggttgtctt
2579521 caagttaggc caagagtccg ctggttcgta caacggttcg tccttcaccg tgaagtacga exon 4
2579581 agataaggat ggcgcactgg tcggaaccat tcactacacc ggtacgaagg aacagagcct
2579641 cgacaagacc atcaacaacg agtacaaggt tgaaggcaat caactggtca agacctcaac
2579701 cctcgaagga gtgacacaca agagatacta caacaaacgc aactgaggtt gttcttgccg
2579761 ctatatggtt gttagttcgg ccaagttttt cctattttcg catcttttgg ctttttctca
2579821 tcattcttcc agtctttatg ttcgcctgtt ctcactgtac tttgctcaag cccatttcca
2579881 gcaacaagtg ctttattcac gtcccagaac cagctttccg ctcgtcgttt acctattcgc
```

```
2579941 ggaagaaatc atccaacatg accaatactg cttctgagcc gataaagtca acgattctcg
2580001 tgctggattc gatttttcga tgtagatact ggacttgact ttgacttgta cggagcgaca
2580061 ccgagcgcag caggaacgca agaagaaaac caagcagata ctttaacccg cttcaaattt
2580121 agagtgggtc tttccaggct gccactgcat tgacccgtgt tttgtttcct gaggtagact
2580181 gatccctctc ctcgaggttt caataggttc gagaacggga aagcgttgta gattacggga
```

***SAHS10* analysis**

SAHS11 and *10*, shown below, are adjacent in the *R. varieornatus* genome and about 5 kilobases from the *SAHS2, 8* cluster. The *SAHS10* annotated start codon lies within a putative intron corresponding to the second intron in *SAHS11* and other *SAHS* genes. *SAHS10* encodes two of the three conserved tryptophans near the N-terminus, while the first tryptophan is replaced with a structurally plausible arginine. Upstream of this coding region, at the position of the splice acceptor found in most *SAHS* genes, a potential splice site is mutated, reminiscent of this site in *SAHS9*.

```
2568721 tcgtctgcca cgtgcacgat gccaggaacg aaaaagcaag ctaggttcag agccaacctg
2568781 ttcatacaca agtgctatcc agctatcctg ctctgcagac ttttaggcta ttctgcacag
2568841 tctccatttc tgttaactgc agttagaacg cctcaattgt catgcggcga tttcttcgca SAHS11
2568901 atcttctcat atttgccagt atgaaccttc ccacacctgt aacgttatgt gtttatgatt
2568961 tttctatttt cgtcctttga atcagaagcc tgactttcca aggataatat aagtaaacga
2569021 aatgtaatta ggtggaccga agggatctag tgttttcccc gtcgcattgg cgaatggctg
2569081 ctgttttgga taattatgat tttcgacgaa tcgataagag cttggctcaa gggactgtat
2569141 tatcccacag cttgatcctt tacaggtgcg gcagtctgcc tggcagagca cgatcctggc exon 2
2569201 catgaggaag gagccgaatg gactggaaaa ccatggctgg gcaaatgggt ttccgttccc Trp codons
2569261 gagaaggacg taaacgtact aaacttcatc acagagatcg gtcagtgcca tgatgcatca
2569321 acttcagccc tcgtggtacg ctcagcggat ggcacgggac attcgaaatg gaatgaattt
2569381 tcacaggtgt cgctgcgagt catccggaac ttcctcccat cgttacggtc ctcgtcaacc exon 3
2569441 attacaaaaa gggcgacgag taccaccaga gactgcgcgt caaggaagta gctgatcttg
2569501 atgatcacga cgtaagtacg atacacggta ctcccttgac ccaggcttaa ggaaggcttc
2569561 ggaccgacgt taacgttaag gaacctgtac tgtagattgt ctacaaactg ggccaagaaa exon 4
2569621 ccaagaacgt ttttaacggt accaccttca gtgttaagta cgatgagaaa gatgacgctc
2569681 tcgtcggaca agtcatgcta ccctcgaaca acgcgactta caagaacgag ttcaaggtcg
2569741 aaggggactt ccttgtcaag gtatcgcagc ccactttgaa ttccgcctca gccattatgt
2569801 cactggtcac atcttttttc gcttgttgca gacctctgac gctcatggaa ttgtccacaa exon 5
2569861 acgatattac aagagacgga actaaatttc aagctggtgc cgaggttcag tctcagattt
2569921 tgtctttgga atcaagctcg cttgcgtgtt tgagttgtcc ccagagtaaa gtcacatgtc
2569981 gttcagttgg cgtgtcgata gaggttcttg tgcttagtct ttagtcgtac aagattttcc
2570041 cgagcagacg ggtggttcag tcggtttcca ctttttttccc acattatccc gcatatctct
2570101 gtatcacaga cgaccactgc cagaaaccgc gccggggcgc ttgaccagat cagatggtga
2570161 gcaaagcagg caatcataat tcatcagcca acctttgaaa ccacgttttt ttcccccgag
2570221 tgtcctaatc ggcgaatgac aatgcttgag gtcgaaaaaa agtgactaca ggctactcac
2570281 aataagggca ggtcaacact atatatgcag gcctctcatc aaaccttcca ttagtttctc
2570341 tacccatcgt actttcttac atagccagct tgaacgatgc atcgatttat ccttcttctc
2570401 gcagtctttt ccggcaagat cgagctcata tcgggatatg gttccttata ctcgggagat
2570461 ggctctgtca tcggttggct ccagccgcta actttccgtg tgcgacgtca ctgcgagaag
2570521 catcggcagg cagaatccgc actgacgcca taagaaagct taagcaagag gagttcggtt
2570581 gtcaagatgc tggatgggaa gctgtctgcg tgttttgttc gttgtgcatg tactcgtact
2570641 gtcatgatgg tgtttcgcgg gtgtggcctt tatctgggcc gccgaagacg ctgttcacga
2570701 agaaggcgta gaacggactg gcaaaccgtg gatgggcaaa tgggtcgccg ttcctgagaa Trp codons
2570761 ggacgaaaat cacgagggac tcaagaaaaa gctccgtgag tacattcgtg ttggcttctg SAHS10
```

```
2570821 accttcattc gtcagttttc ctcgacaatc gtcgatgtga tgtatgagcc accatggga “start”
2570881 tgcgatctgc agatatcccc ttgagtcacc cgcatctgaa acacaacaac agagtgtggg
2570941 ttaacaccta caagaaggga gacgaatacc accacaagat tattatcaag gaagccggct
2571001 ataccaatga tgtacgtatg cgaagccatt atgattatgc aaactgccga acgctgcatg
2571061 gctttaatct ttccacacag tacgtgcgta ctgagatctc ttgcatacct gagactgatt
2571121 ttcaggttgt cttcaagctg ggtcaagagt ccgccggctc gcataacggc tcatctttca
2571181 gcttgaagta cgaagacaag gatggcgcct tggtcggcac cgtccatcgc accggcacca
2571241 aggaacagcc cctggacaag acgatcaaca acgtcttcaa gctcgagggt gaccatttag
2571301 ttatgacctc caccatcgac ggagtaacca tgaaacgcta ctacaagaca cgaacgtgaa
```

SAHS12 analysis

In the annotation of Scaffold 1 of the *R. varieornatus* genome, the ORF upstream of *SAHS12* (*RvY_02619-1*) encodes a protein whose C-terminal region aligns well with the N-terminus of the mature SAHS proteins. A splice donor is present near the end of this sequence that corresponds to intron 2 of *SAHS2* and other well-annotated *SAHS* genes.

This region thus may encode a large protein with the sequence MTQPMSFAQCSADRGKHSGTTIWTLLRIYLACQKAILRSKLRLRAPLFPTVEPNPAPIQNAAPAPSAAQRRRNFAASHAANVDLPGSVWHGETWGDQHDPPNRLAADVDNFDWRSKFWLGKWSSIPEKDQNLEAYLAVMgvdMNHPNMKKDQPVTLQTFKKGDKYHHKIVVEEAGYINDVIFRLGRETPGSYNGQQITVNYEEQGGALVGTVKYPAHNKVIHNTYEMDGQNLAKTSECEGVVHKRWYNKQQN, where the black amino acids are from *RvY_02619-1*, the “gvd” is glycine-valine-aspartate arising from the splice junction and segment upstream of the annotated SAHS12 start codon, and the brown amino acids are from SAHS12 as annotated in the Scaffold 1 annotation.

```
2002141 gatgagcaac aagtaatggc tcggtaccag ctaagacctg aggcaaaatc aacgcggatg
2002201 gatgcagatc tatttcagtg aacaaaggac ggaagatttc tgatgggagg aaaagtagta
2002261 cagtgtaata ataatccgag aaagaaaatc aaattctgac tttcagagtc atctgagatg start of
2002321 actcaaccga tgagtttcgc acagtgctcg gcggaccgcg gtaaacattc cggtaccacg RvY_02619
2002381 atctggacgc tgctgcgaat atacttggcc tgccaaaaag ccatccttcg atcaaaatta
2002441 aggctacgag gtaaacgtca caattcggac gggccaattt tctcgtcaca attcgggcgg
2002501 ctcgaaaagt tgatacgtta tttgctacgc tgtattgatt gatcaccgta gatcggttga
2002561 tacaacttta ggctttttaa cattgcgcat acccctcttc ctcccaccgc tgactccgca
2002621 actctttgac atctttcgat atctcaacct atcacaagag atagaagaga ggatgttcta
2002681 ccctgggaag caaaaactag gcagtttgga gcagaaacag ctgagataca gaccgcccaa
2002741 attgtgacga gaaaattggc ccgcccgaat tgggacgttt acctcgtaac gattatgtgt
2002801 tttgtcttct aatcagctaa tcaagtcatt ggttttcatg aggattatgt tgatgcatga
2002861 ttcctcgagc ttttgacagg ccgtaccgtg cttcacggag tgtttacccg ccgaatactg
2002921 ccaatgagtc attagaagca ccaggattgg tctgttactc tgtcctattc aaatcaggcg
2002981 aagcgggaga atgacgaagc ggatactgac gcttagcctt cgcatggtct gttgtttcgg
```

2003041 cttgcaccgt tccatccaga ttttagggtg agcaaccgtc agcaacgtgg ccgtcctggc
2003101 aaagtctttt cctgtcctca ccttcagctc ctttatttcc cactgtggag cctaatccgg exon 2 of
2003161 cacccatcca gaacgcagct cccgctccat cggctgccca acgtcgtcgg aactttgcag *RvY_02619*
2003221 ccagtcatgc cgccaatgtc gacctgccag gctccgtttg gcacggggaa acctggggag
2003281 atcaacatga tccgccgaac cgattagcag ctgatgtcga caacttcgac **tgg**agatcga **Trp codons**
2003341 aattc**tgg**ct gggcaag**tgg** agctctatcc cagagaagga tcaaaatttg gaggcttacc
2003401 ttgctgtcat gggtaagccg gagacttacg cttgattgac tgtcattgat tgactggctt
2003461 ccaacttccg cttccgggtt caacaggtgt cgac**atg**aac catcccaaca tgaagaagga *SAHS12*
2003521 tcaacccgtt acacttcaga ccttcaagaa gggtgacaag taccatcata agatcgtggt “start”
2003581 cgaggaagcc ggctacatta acgatgtaag tttgatggac ctggcagctt tcttccagcc
2003641 ggatgtcatg ttgctttatc cgacgcgaat gtagtaacgc ctttcatatt actccatgtt
2003701 gtgccgtaat ggagaagcgt ggcgataaca tgcgtgtgat acgcatggta ttccggacag
2003761 ggaggcctat gtgtcctttt attgttattc caggttattt tccgcctcgg ccgagagact
2003821 cccggatctt ataacggtca acagatcact gtcaactatg aggaacaagg cggtgctttg
2003881 gtgggtaccg tcaagtatcc cgcccataac aaggtcatcc ataataccta cgagatggat
2003941 gggcagaatc tggccaaggt atcaaacctt acttcctctt ttgcagcttt tttcctggaa
2004001 acgccggtct gacaatttgc tgacagcggt gctcgtttgt tgcagacttc cgaatgtgag
2004061 ggtgtcgttc acaagcgctg gtataacaag cagcaaaac**t** **ga**agcctgtc gcctccatta
2004121 attgtgatag ttttgccttc gagttacgat tcctcatgaa agtgcttttc atgtatgtct
2004181 gccattttaa ctaactgtac cagatgttga tttacggttt tggatagctg cagtattcct
2004241 tcagagaact ttgcgatgca acgaaccatg ttccttcttt gtccactgtg aatacgatgt
2004301 gctgcgatct actatggaag cactgcctac gtagagaaaa ccgaaaatgt cctgcctcag
2004361 aactagtttc cagtttccta gacatttcga caccctccag tatctttctc gcttagggtg
2004421 tcgcacgaac agaaactaca gttctaactg cgcctttcgg cggctgactt cgcattcgaa

Supplementary Computational Simulation Methods

Overview. The goal of these simulations was to provide potential insight into the transition of SAHS1 from its primarily beta-sheet structure to some other structure that it might adopt upon desiccation. To this end, we generated an AlphaFold prediction of the structure and interactions of a set of three SAHS1 proteins, and then simulated the behavior at a very high temperature, 550 Kelvin, for 1 microsecond. This simulation indicated that alpha helices can transiently form and disappear, and that most of the amino acids are converted to a coil conformation as defined by their phi/psi backbone angles. The structures do not reach an equilibrium state.

The simulation conditions do not completely replicate the biological process that we are trying to understand, which likely plays out over hours instead of microseconds, and which likely involves rather gradual withdrawal of water, Brownian collisions with unrelated proteins (*in vivo*), and denaturation that may be driven by loss of material within the cavity of SAHS1 instead of by high temperatures. For example, we performed circular dichroism measurements after dialyzing our protein overnight into the denaturing agent trifluoroethanol (TFE). The simulation we performed was designed to be exploratory and might represent part of the denaturation process and illustrate how alpha helices might be nucleated from a disordered structure, but the other aspects of desiccation are not easily simulated with current technology.

AlphaFold structure prediction. We used Alphafold2 [7]. The starting structure predicted for the trimer is provided as a supplementary file.

Predicted structures were generated for 1, 2 ,3, 4, 8, and 16 SAHS1 molecules, and various structural complexes with varying degrees of plausibility were revealed. The choice of

using three SAHS1 proteins for simulation was to strike a balance between capturing protein-protein interactions while not building an inefficient simulation system for long timescales.

In addition, the AlphaFold structure for three copies of SAHS1 predicted that an N-terminal tail of this protein would fit into the cavity of another SAHS1 protein. This is illustrated in Figure S10.

Comparison of the three different SAHS1 proteins in the simulation. To save on computational time, rather than performing separate simulations we performed a single simulation with three copies of the SAHS1 protein present in a water bath. The results, illustrated in Figure S8, were similar for each copy of SAHS1 and indicated that the amino acid psi/phi angles in each protein went from a ~50% to ~0% beta conformations within 20-40 nanoseconds, to almost completely coiled conformations in the same period, and showed a slight increase in alpha helical conformations that peaked at about 40 nanoseconds and was roughly constant thereafter.

Simulation details.

number of simulations	1 simulation with 3 protein copies
simulation box dimensions	100 Å cube
total number of atoms	124,411
total number of water molecules	39,038
salt concentration	74 x Cl- ions and 83 x Na+ (neutral charge)
Amino acid protonation state	Typical for amino acid side chains at pH 7.5

Solvation box details. A 100 Å simulation box with TIP3 water as solvent was generated using Ambertools and the amber forcefield (ff14SB) (https://ambermd.org/doc12/AmberTools13.pdf; https://ambermd.org/Manuals.php). Na^+ and Cl^- ions were added to achieve a neutral charge. Simulations were performed using Openmm 7 (http://docs.openmm.org/7.7.0/developerguide/), periodic boundary conditions, and a Particle-

Mesh Ewald with a cutoff of 1*nanometers and an Ewald error tolerance of 0.0005. A Monte Carlo barostat was used at 1-atmosphere pressure and an interval of 25 using the Langevin Integrator. Following equilibration at 310 K, the simulation was extended from a restart checkpoint, and velocities reset to the 550 K temperature.

Secondary structure assignments. The Python package MDTraj was used. Specifically, the protein secondary structure (DSSP) secondary structure assignments function (https://mdtraj.org/1.9.4/api/generated/mdtraj.compute_dssp.html). This function implements the assignment based on the reference [8].

The simplified version was executed, which groups the secondary structure into helical, strand, and coil. “Helical” includes Alpha helix, 3-helix (3/10 helix), and 5 helix (pi helix). “Strand” includes residues in isolated beta-bridge and extended strands, participating in beta ladders. The coil includes hydrogen-bonded turns and bends.

Supplementary References

1. Malki, A. *et al.* Intrinsically Disordered Tardigrade Proteins Self-Assemble into Fibrous Gels in Response to Environmental Stress. *Angew. Chem. Int. Ed.* **61***,* e202109961 (2022).
2. Yagi-Utsumi *et al.* Desiccation-induced fibrous condensation of CAHS protein from an anhydrobiotic tardigrade. *Sci. Rep.* **11**, 21328 (2021).
3. Yamaguchi, A. *et al.* Two novel heat-soluble protein families abundantly expressed in an anhydrobiotic tardigrade. *PLoS One* **7**, e44209; 10.1371/journal.pone.0044209 (2012).
4. Fukuda, Y., Miura, Y., Mizohata, E. & Inoue, T. Structural insights into a secretory abundant heat-soluble protein from an anhydrobiotic tardigrade, Ramazzottius varieornatus. *FEBS Lett.* **591**, 2458-2469 (2017).
5. Fukuda, Y. & Inoue, T. Crystal structure of secretory abundant heat soluble protein 4 from one of the toughest "water bears" micro-animals Ramazzottius varieornatus. *Protein Sci.* **27**, 993-999 (2018).
6. Hashimoto T. *et al.* Extremotolerant tardigrade genome and improved radiotolerance of human cultured cells by tardigrade-unique protein. *Nat. Commun.* **7**, 12808; 10.1038/ncomms12808 (2016).
7. Jumper, J. *et al.* Highly accurate protein structure prediction with AlphaFold. *Nature* **596**, 583-589 (2021). https://github.com/google-deepmind/alphafold
8. Kabsch, W. & Sander, C. Dictionary of protein secondary structure: pattern recognition of hydrogen-bonded and geometrical features. *Biopolymers: Original Research on Biomolecules* **22**, 2577-2637 (1983).
