# Supplementary material for: Tardigrade secretory proteins protect biological structures from desiccation
Source: Commun Biol. 2024 May 25;7:633. doi: 10.1038/s42003-024-06336-w (PMC11127935; doi:10.1038/s42003-024-06336-w)
Supplement: Supplementary file 3 — Description of Supplementary Materials [file 42003_2024_6336_MOESM3_ESM.docx]

**Description of Additional Supplementary Files**

**File name:** Supplementary Data 1

**Description:** the source data behind the graphs in the paper

**File name:** Supplementary Data 2

**Description:** the PDB files for the MD simulations performed in the paper
